# Supplementary material for: Precise Quantification of Molybdate In Vitro by the FRET-Based Nanosensor ‘MolyProbe’
Source: Molecules. 2022 Jun 8;27(12):3691. doi: 10.3390/molecules27123691 (PMC9228995; doi:10.3390/molecules27123691)
Supplement: Supplementary file 1 [file molecules-27-03691-s001.zip › molecules-1701398-supplementary.pdf]

molecules

MDPI

Supplementary Materials

Precise Quantification of Molybdate In Vitro by the FRET-Based Nanosensor 'MolyProbe'

Kevin D. Oliphant [1], Marius Karger [1], Yoichi Nakanishi [2] and Ralf R. Mendel [1,*]

[1] Department of Plant Biology, Braunschweig University of Technology, 38106 Braunschweig, Germany; k.oliphant@tu-braunschweig.de (K.D.O.); marius.karger@tu-braunschweig.de (M.K.)
[2] Department of Applied Biosciences, Graduate School of Bioagricultural Sciences, Nagoya University, Nagoya 464-8601, Japan; nakanish@agr.nagoya-u.ac.jp
* Correspondence: r.mendel@tu-braunschweig.de

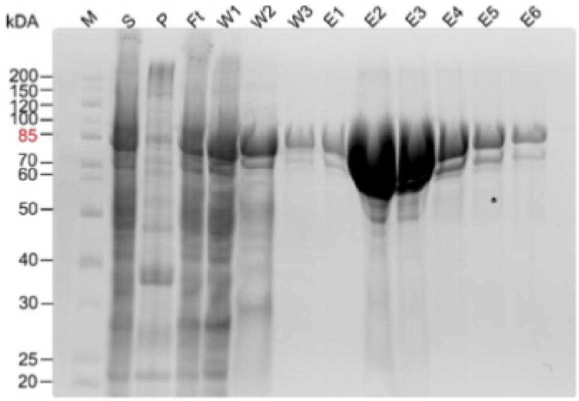

Figure S1. MolyProbe-Strep affinity purification. SDS-PAGE of C-terminal Strep-tagged MolyProbe. The gel was loaded from left to right as follows: Ladder (M) (Pierce Unstained Molecular weight ladder, Thermo Fischer Scientific), supernatant (S), pellet (P), flowthrough (Ft), washing steps (W1-W3), elution fractions (E1-E6).

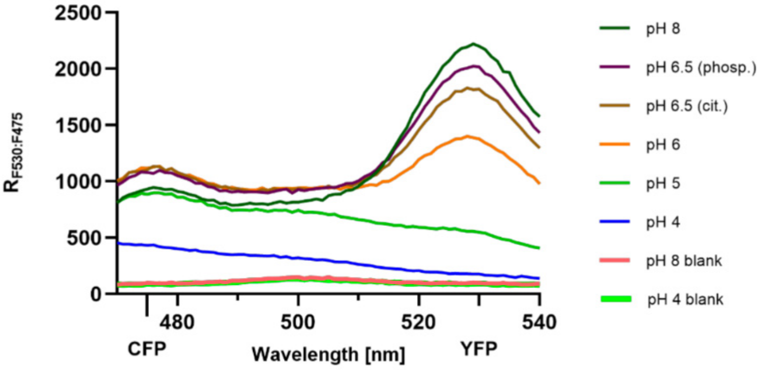

Figure S2. pH-dependent MolyProbe emission. Emission spectrum from 470 to 540 nm of 30 nM MolyProbe supplemented with 1 µM molybdate, excited at 430 nm. Emission spectrum was measured from 470 to 550 nm in 100 µl sample volume. pH 4 to 6.5 was in 50 mM citrate buffer, pH 6.5 to 8 in 50 mM phosphate buffer.

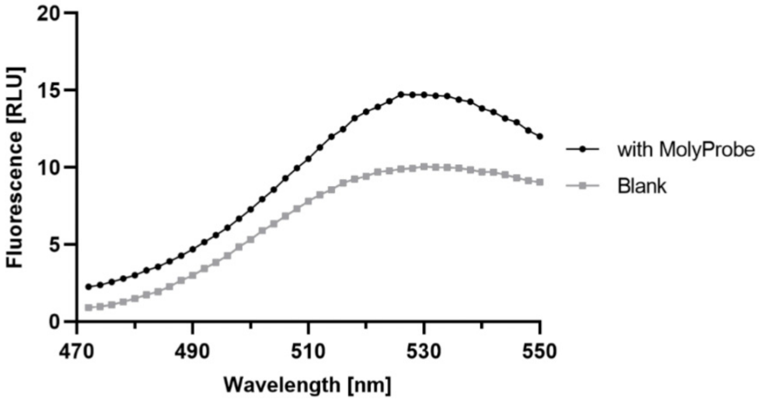

Figure S3. Interference of *N. crassa* autofluorescence. The spectra of FRET assay measurements with 40-fold diluted N. crassa cell extracts. The black curve shows the emission spectrum of a *N. crassa* sample with 20 nM MolyProbe. The grey curve shows the same *N. crassa* sample without added MolyProbe, serving as a blank. The pictured sample was supplemented with 1 mM molybdate. Excitation was at 430 nm, and the emission spectrum was measured from 470 to 550 nm.

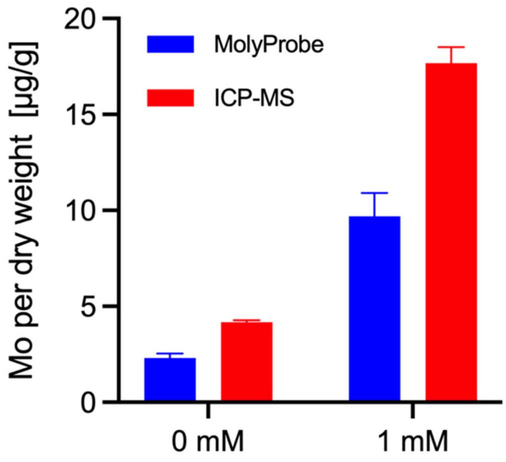

Figure S4. Comparison of Mo measurements by the MolyProbe assay with ICP-MS. *Neurospora crassa* was cultured and harvested as described in the Materials section. The cultures were supplemented with or without 1 mM molybdate. The inductively coupled plasma mass spectrometry (ICP-MS) results were analyzed with a standard nitric acid extraction protocol using an Agilent 7700 Series ICP-MS (Agilent Technologies). For calibration, a standard curve of sodium

molybdate (1–20 mg/l, Fluka) was used. Crude extract and standards were mixed automatically using rhodium ($Rh(NO_3)_3$) as internal standard. Data collection and processing were carried out using the MassHunter work station software. The spectra of FRET assay measurements were taken with 40-fold diluted *N. crassa* cell extracts. Excitation was at 430 nm, and the emission spectrum was measured from 470 to 550 nm. ICP-MS and MolyProbe measurements n = 3.
